# DNA methylation profile of Aire-deficient mouse medullary thymic epithelial cells

**DOI:** 10.1186/1471-2172-13-58

**Published:** 2012-11-02

**Authors:** Guoying Wu, Keiji Hirabayashi, Shinya Sato, Nobuko Akiyama, Taishin Akiyama, Kunio Shiota, Shintaro Yagi

**Affiliations:** 1 Laboratory of Cellular Biochemistry, Department of Animal Resource Sciences /Veterinary Medical Science, Graduate School of Agricultural and Life Sciences, The University of Tokyo, 1-1-1, Yayoi, Bunkyo-ku, Tokyo, 113-8657, Japan; 2 Division of Cellular and Molecular Biology, Institute of Medical Science, The University of Tokyo, 4-6-1, Shirokane-dai, Minato-ku, Tokyo, 108-8639, Japan

**Keywords:** Medullary thymic epithelial cells, Aire, T-DMR

## Abstract

**Background:**

Medullary thymic epithelial cells (mTECs) are characterized by ectopic expression of self-antigens during the establishment of central tolerance. The autoimmune regulator (Aire), which is specifically expressed in mTECs, is responsible for the expression of a large repertoire of tissue-restricted antigens (TRAs) and plays a role in the development of mTECs. However, Aire-deficient mTECs still express TRAs. Moreover, a subset of mTECs, which are considered to be at a stage of terminal differentiation, exists in the Aire-deficient thymus. The phenotype of a specific cell type in a multicellular organism is governed by the epigenetic regulation system. DNA methylation modification is an important component of this system. Every cell or tissue type displays a DNA methylation profile, consisting of tissue-dependent and differentially methylated regions (T-DMRs), and this profile is involved in cell-type-specific genome usage. The aim of this study was to examine the DNA methylation profile of mTECs by using Aire-deficient mTECs as a model.

**Results:**

We identified the T-DMRs of mTECs (mTEC-T-DMRs) via genome-wide DNA methylation analysis of *Aire*^−/−^ mTECs by comparison with the liver, brain, thymus, and embryonic stem cells. The hypomethylated mTEC-T-DMRs in *Aire*^−/−^ mTECs were associated with mTEC-specific genes, including *Aire*, *CD80*, and *Trp63*, as well as other genes involved in the RANK signaling pathway. While these mTEC-T-DMRs were also hypomethylated in *Aire*^+/+^ mTECs, they were hypermethylated in control thymic stromal cells. We compared the pattern of DNA methylation levels at a total of 55 mTEC-T-DMRs and adjacent regions and found that the DNA methylation status was similar for *Aire*^+/+^ and *Aire*^−/−^ mTECs but distinct from that of athymic cells and tissues.

**Conclusions:**

These results indicate a unique DNA methylation profile that is independent of Aire in mTECs. This profile is distinct from other cell types in the thymic microenvironment and is indicated to be involved in the differentiation of the mTEC lineage.

## Background

Medullary thymic epithelial cells (mTECs) are highly involved in the establishment of central tolerance by ectopically expressing a variety of tissue-restricted antigens (TRAs) [1]. Deficiency of the autoimmune regulator, Aire—which regulates a large pool of genes in mTECs—results in autoimmune polyglandular syndrome in humans and autoimmunity in mice [2]. There are estimated to be hundreds to over one thousand TRAs that are activated by Aire in mTECs, based on microarray data from the transcriptomes of wild-type and Aire-deficient mTECs [3,4]. However, numerous TRAs are expressed in mature mTECs in an Aire-independent pattern [3,4]. Moreover, single-cell PCR analysis with sorted mTECs demonstrated that approximately 70% [5], or even fewer [6], of mature mTECs express Aire, and TRAs are expressed in Aire-negative mTECs [5]. These findings indicate that Aire is necessary, but not sufficient, for the expression of TRAs in mTECs.

The role of Aire in the maturation of mTECs has been indicated by using *Aire*-deficient mice, which exhibit contracted compartments in the thymic medulla and an increased number of medullary cells positive for p63, whose expression is diminished in Aire-positive mTECs [6-9]. The role of Aire in the differentiation of mTECs has been reviewed by Matsumoto [10]. However, the existence of the medullary compartment and the increased number of a small subset of K5^−^K8^+^ cells—considered to represent end-stage and terminally differentiated mTECs—in the Aire-deficient thymus indicate a limited role of Aire in the lineage determination of mTECs, in which the RANK signaling pathway is involved [11,12]. Thus, Aire-deficient mTECs may represent a suitable model to study not only Aire-independent TRA expression but also the fundamental regulation system that governs cell lineage determination of mTECs.

Genome-wide analysis of DNA methylation has indicated that a specific DNA methylation profile exists for every cell or tissue type. This profile consists of numerous tissue-dependent and differentially methylated regions (T-DMRs) at cytosine residues in CpG dinucleotides (CpGs), which are differentially methylated among distinct cells and tissues [13]. DNA methylation profiles change during cell differentiation, reflect developmental similarity among cell lineages [14,15], and illuminate cell- or tissue-specific gene expression profiles [16-18]. These findings suggest that DNA methylation profiles are involved in the establishment and maintenance of the regulatory system for cell- or tissue-type-specific genome use, and could therefore be used to identify types of cells and tissues.

The DNA methylation profile responsible for the phenotype of mTECs is still largely unknown, although a limited number of genes, including *Aire* and 2 Aire-independent TRA genes, *Csn2* (Csnb) and *Gad1* (Gad67), are unmethylated in their promoter regions [19-21]. To explore the genome-wide DNA methylation of mTECs, we applied a microarray-based screening system, T-DMR profiling with restriction-tag-mediated amplification (D-REAM) [16], to UEA1^+^CD45^−^ mTECs that were isolated from *Aire*-deficient (*Aire*^−/−^) mice [22]. We identified T-DMRs in mTECs (mTEC-T-DMRs) and compared the DNA methylation status at mTEC-T-DMRs in *Aire*^+/+^ and *Aire*^−/−^ mTECs, UEA1^−^CD45^−^ thymic stromal cells, and athymic cell and tissues.

## Results and discussion

### mTEC-T-DMRs identified in *Aire*^−/−^ mTECs are associated with mTEC-specific genes

**Figure 1:**
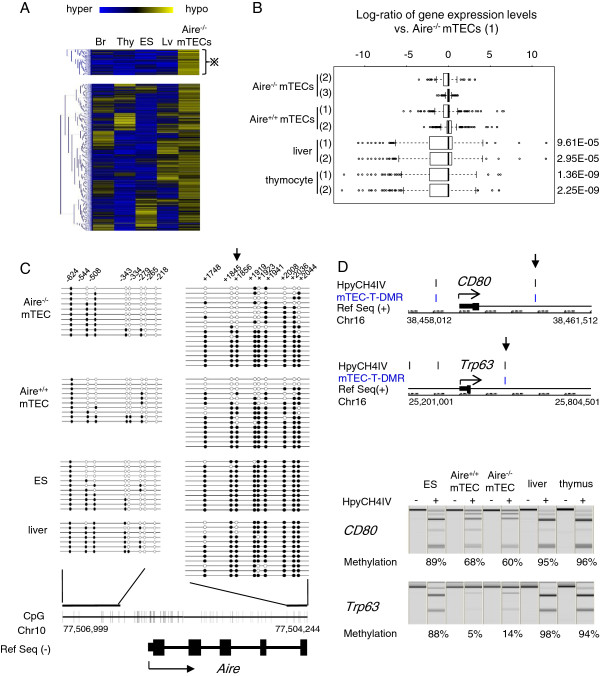
**mTECany2-T-DMRs correlate with gene expression in mTECs and associate with marker genes of mTECs. A.** Hierarchical clustering of D-REAM scores corresponding to mTECany2-T-DMRs identified by comparison of *Aire*^−/−^ mTECs with the brain, thymus, liver, and ES cells by Pearson’s correlations. Among mTECany2-T-DMRs, those hypomethylated in *Aire*^−/−^ mTECs in comparison with all of the reference cells and tissues were defined as mTECu-T-DMRs (indicated by asterisk). Br, brain; Thy, thymus; Lv, liver. **B.** Relative expression levels of genes associated with mTECu-T-DMRs in proximal regions (−6 to +2.5 kb) from TSS. The horizontal axis of the boxplot represents the log ratios of gene expression levels relative to *Aire*^−/−^ mTECs. The numbers in round brackets indicate independent gene expression profiles for each type of cell or tissue (Additional file 5: Table S2). The *p*-values of *t*-tests showing significant differences in relative expression levels are shown. **C.** Bisulfite sequencing of regions around the TSS of *Aire*. The arrowhead indicates the position of the mTECu-T-DMR. The regions upstream (−674 to −191 bp) and downstream (+1672 to +2082 bp) of the TSS were analyzed independently using bisulfite sequencing. Open and closed circles represent unmethylated and methylated CpGs, respectively. Positions of CpGs are shown as vertical bars above the genomic structure (shown at the bottom of the panel), and the boxes indicate exons. **D.** COBRA analysis of mTECu-T-DMRs associated with *Cd80* and *Trp63*. Positions of HpyCH4IV sites and mTECu-T-DMRs are shown above the genomic sequences within −1 to +2.5 kb of the TSS. Electropherograms represent the bisulfite PCR products after digestion with HpyCH4IV. Hypomethylated fragments were resistant to HpyCH4IV digestion (+). The levels of DNA methylation (%) are shown under each lane of the electropherograms.

Thymic stromal cells from *Aire*^−/−^ mice and their *Aire*^+/+^ littermates were fractioned into 2 populations: UEA1^+^CD45^−^ (mTECs) and UEA1^−^CD45^−^ (control thymic stromal cells). In this study, we use the term “stromal” to refer to UEA1^−^CD45^−^ cells, which are a mixture of keratin-expressing epithelial cells, including cortical TECs (cTECs), and keratin-negative mesenchymal cells such as fibroblasts, connective tissue cells, and endothelial cells [23]. Predominant expression of *Aire*, *Cd80*, *Krt5*, *Krt8*, *Krt14*, and *Foxn1* in the UEA1^+^CD45^−^ population (detected by RT-PCR) indicated enrichment of mTECs in this fraction (Additional file 1: Figure S1). To elucidate the characteristics of genome-wide DNA methylation in mTECs, we compared *Aire*^−/−^ mTECs with the liver, brain, thymus, and embryonic stem (ES) cells by D-REAM. mTEC-T-DMRs, which were differentially methylated as compared with the other cells and tissue types, were screened as genomic fragments that exhibited significantly different microarray scores after digestion by the methylation-sensitive restriction enzyme, HpyCH4IV (Additional file 2: Figure S2). Among the mTEChypo-T-DMRs, which were hypomethylated in *Aire*^−/−^mTECs, we designated those that exhibited scores higher than all or any 2 of the reference cells and tissues as mTEC-unique T-DMRs (mTECu-T-DMRs) or mTECany2-T-DMRs, respectively (Figure 1A, Additional file 3: Table S1).

The mTECany2-T-DMRs mapped to within 8.5-kb regions around the transcription start sites (TSSs) of 3081 ENSTs (Ensembl transcripts; mm9) (Additional file 3: Table S1). The distribution of mTECany2-T-DMRs exhibited a similar pattern to that observed in previous analyses of somatic tissues and ES cells [16,17], displaying a peak of T-DMRs at the 3'-downstream regions of TSSs (Additional file 4: Figure S3). Hypomethylated signals in these regions are often associated with cell- and tissue-specific highly expressed genes [16,17].

We analyzed the expression of ENSTs associated with mTECany2-T-DMRs in mTECs using the gene expression profile of CDR1^int^B7-1^hi^ CD45^−^ mTECs from the Gene Expression Omnibus (GEO) database, and compared with those of mouse liver, and CD4^+^CD8^+^ thymocytes as a representative of the thymic cells (Additional file 5: Table S2). The differences in expression levels of genes associated with mTECany2-T-DMRs were relatively small between *Aire*^−/−^ and *Aire*^+/+^ mTECs as compared with those between mTECs and the liver or thymocytes, indicating that the expression patterns of genes associated with mTECany2-T-DMRs in mTECs are not affected by functional Aire (Additional file 6: Figure S4). High gene expression in mTECs was more prominent for genes associated with mTECany2-T-DMRs in the −1.5 kb upstream and +2.5 kb downstream (proximal) regions of TSSs. Among these genes, those associated with proximal mTECu-T-DMRs showed significantly higher expression in mTECs compared with both the liver and thymocytes (Figure 1B). These data indicate that mTEC-specific genes are likely to be associated with mTECu-T-DMRs, and methylation may be responsible for gene silencing in Aire non-expressing tissues and cells. Indeed, we found that among the mTECu-T-DMR–associated genes, some had been previously reported as mTEC-specific genes, of which the majority showed Aire-independent expression in mTECs (Additional file 7: Table S3). Moreover, marker genes of mTECs, including *Aire*, *Cd80*, and *Trp63*, were found to be associated with proximal mTECu-T-DMRs.

We analyzed the DNA methylation status around the mTECu-T-DMR of *Aire* which is located downstream of its TSS, and the upstream region by using bisulfite sequencing (Figure 1C). In the −624 to −218 bp upstream region, the liver, which represents an Aire non-expressing tissue, showed a methylated status comparable with *Aire*^+/+^ and *Aire*^−/−^ mTECs as well as with ES cells, which express Aire [24]. The promoter regions of human *AIRE* (−295 to +65 bp) and mouse *Aire* (−287 to +133 bp) are both associated with a CpG island (>50% CpG content) and are unmethylated in mTECs [19,20]. Since Aire-negative cTECs and several extrathymic tissues were also found to be unmethylated at this region [20], hypomethylation of the promoter is considered to be necessary, but not sufficient, for expression. In contrast, *Aire*^+/+^ and *Aire*^−/−^ mTECs, but not ES cells, exhibited a hypomethylated status at the 9 CpGs in the region between +2082 and +1672 bp from the TSS, including the CpG corresponding to the mTECu-T-DMR. Among the tissue-specific genes carrying CpG islands within 1 kb of their TSSs, hypomethylated T-DMRs tend to be located downstream of TSSs [16,17]. Thus, the mTEC-TDMR for *Aire* located 3'-downstream of the *Aire* TSS could serve as an epigenetic marker to distinguish mTECs from Aire-expressing ES cells, in which the expression level of *Aire* was significantly less than that in the thymus and mTECs (Additional file 1: Figure S1).

The mTECu-T-DMRs associated with CD80 and p63 (*Trp63*) were analyzed with combined bisulfite restriction analysis (COBRA). Both *Aire*^+/+^ and *Aire*^−/−^ mTECs exhibited a hypomethylated status compared with the liver, thymus, and ES cells (Figure 1D). Notably, the mTECu-T-DMR of p63 was found to be associated with ΔNp63, the short isoform of p63, which is essential for maintenance of the progenitor status of thymic epithelial cells [25] and not expressed in Aire-positive mTECs [7-9]. Hypomethylation at this T-DMR in *Aire*^+/+^ mTECs suggests that DNA methylation does not determine the repression of p63 in *Aire*^+/+^ mTEC cells.

### mTECany2-T-DMRs are specific to mTECs, as compared with the other thymic stromal cells

**Figure 2:**
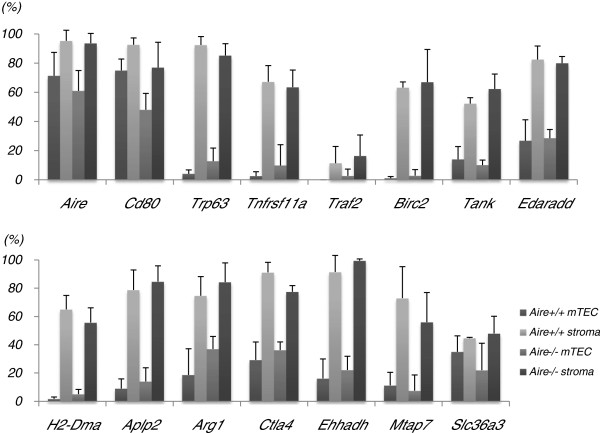
**UEA1**^**+**^**CD45**^**−**^**mTECs and UEA1**^**−**^**CD45**^**−**^**thymic stromal cells display distinct DNA methylation levels at mTECany2-T-DMRs.** DNA methylation levels at the mTECany2-T-DMRs in mTECs and stromal cells isolated from *Aire*^+/+^ and *Aire*^−/−^ littermate mice, as detected by COBRA. The levels of DNA methylation at CpGs within the HpyCH4IV site are indicated by the vertical axis. For *Aire* and *CD80* genes—containing 2 HpyCH4IV sites inside the investigated region—the DNA methylation rate was calculated when any 1 of the CpGs was methylated. For the remaining genes, the levels represent DNA methylation levels at a single CpG. Bar graphs indicate means ± standard errors of the data from at least 3 independent bisulfite PCRs using independently isolated cells from 3 *Aire*^+/+^ mice and 3 *Aire*^−/−^ mice.

We compared the DNA methylation levels of 15 selected genes—which were shown to be associated with mTECany2-T-DMRs—between the mTECs and stromal cells from *Aire*^+/+^ and *Aire*^−/−^ mice by COBRA (Figure 2). In contrast to the hypomethylated status in mTECs, *Aire*, *Cd80*, and *Trp6*3 were hypermethylated in stromal cells. Some genes that associated with mTECany2-T-DMRs were found to be involved in the RANK/RANKL signaling pathway, which stimulates the downstream NF-κB pathway and is required for the development of mTECs [11,12,26] (Additional file 8: Figure S5). For example, *Tnfrsf11a* (RANK) was hypomethylated in mTECs but hypermethylated in stromal cells. *Traf2*, the gene downstream of RANK, was hypomethylated not only in mTECs but also in the stroma, while its downstream genes, *Tank*, *Birc2*, and *Edaradd* demonstrated similar methylation patterns to *Tnfrsf11a*. We also found that *H2-Dma* (H2-Dm), which exhibited 2 mTECu-T-DMRs surrounding its second exon—a region essential for antigen presentation by MHCII [27]—was hypomethylated in mTECs and hypermethylated in the remaining stromal cells. In addition to these genes, similar methylation patterns were observed at mTECany2-T-DMRs associated with genes exhibiting tissue-specific expression patterns, such as *Aplp2* (adipose tissue and brain), *Arg1* (liver), *Ctla4* (Treg), *Ehhadh* (kidney and liver), *Mtap7* (lens), and *Slc36a3* (testis). Importantly, these methylation patterns were observed in both *Aire*^−/−^ and *Aire*^+/+^ mTECs, demonstrating that a DNA methylation pattern distinct from other thymic stromal cells is maintained in *Aire*^+/+^ mTECs.

### *Aire*^+/+^ and *Aire*^−/−^ mTECs show similar DNA methylation levels at mTEC-T-DMRs

**Figure 3:**
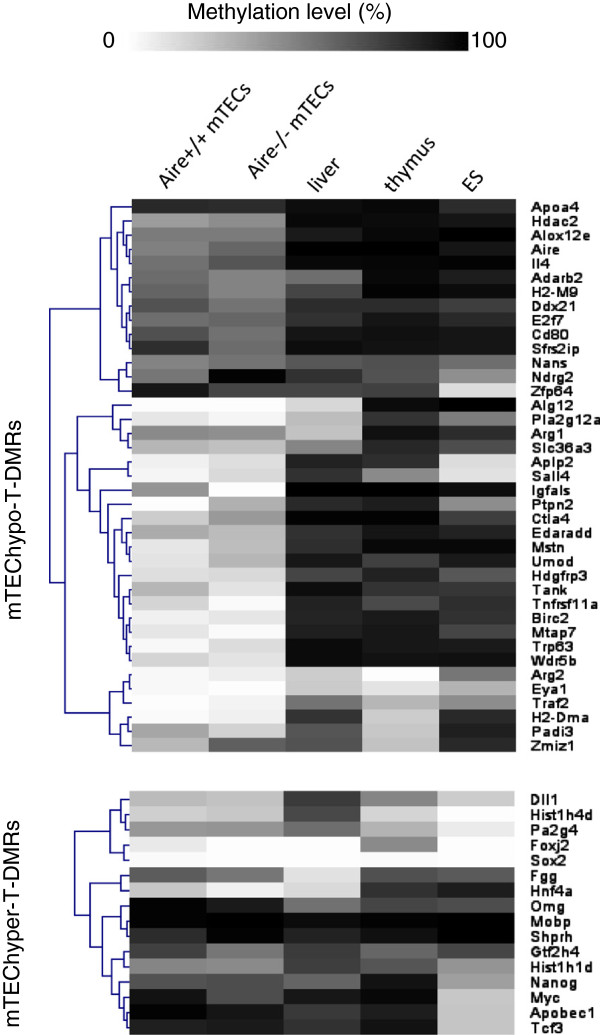
***Aire***^**+/+**^**and*Aire***^**−/−**^**mTECs show similar DNA methylation profiles.** Hierarchical clustering with Euclidean distance of DNA methylation levels estimated by COBRA at 39 mTECany2-T-DMRs and adjacent T-DMRs (upper panel) and 16 previously identified tissue T-DMRs [16,17], which overlap with mTEChyper-T-DMRs (lower panel, Additional file 3: Table S1). DNA methylation levels from 0% to 100% are represented as the continuous gradation from white to black. Each row represents a different gene associated with T-DMRs.

To further compare the DNA methylation status between *Aire*^+/+^ and *Aire*^−/−^ mTECs, we used COBRA to analyze 55 loci, including mTEC-T-DMRs and HpyCH4IV sites in the vicinity and some previously identified T-DMRs of other cell and tissue types [16,17] (Figure 3, Additional file 9: Table S4). Hierarchical clustering of DNA methylation levels at these mTEC-T-DMRs showed that *Aire*^+/+^ and *Aire*^−/−^ mTECs display the most similar pattern (less than 20% difference in DNA methylation) compared with the liver, thymus, and ES cells. DNA methylation levels at mTEChypo-T-DMRs in both mTECs showed a unique or common hypomethylated status compared with the reference cell and tissues (Figure 3, upper panel), as revealed by D-REAM analysis (Figure 1A). Genes associated with T-DMRs characteristic of other cell and tissues were hypermethylated in mTECs (Figure 3. lower panel). These data indicate that the presence of Aire does not greatly change DNA methylation levels in mTECs.

Among the analyzed genes, *Fgg* (fibrinogen gamma chain), which is a liver-specific gene that is expressed in mTECs in an Aire-dependent pattern [3,4], was hypermethylated in both *Aire*^+/+^ and *Aire*^−/−^ mTECs, similar to its status in ES cells and the thymus. The gene expression of TRAs in mTECs occurs in specific subpopulations and in a stochastic manner [1,2]; therefore, DNA methylation changes may occur at specific loci in an Aire-dependent manner in subpopulations of mTECs that express a specific TRA.

During thymic organogenesis, Aire is expressed at E16.5 [28], while the stromal cells of the medulla and cortex are separated at embryonic day 13 (E13) [29]. In the absence of Aire, mTECs survive Aire-induced apoptosis, reach a terminally differentiated stage [10], and maintain the ability to express TRAs [3,4]. Thus, the similar DNA methylation status at T-DMRs in *Aire*^+/+^ and *Aire*^−/−^ mTECs suggests the existence of an Aire-independent DNA methylation profile that serves as an intrinsic system to determine the phenotype of mTECs.

## Conclusions

In this study, we demonstrated the DNA methylation profile for *Aire*^−/−^ mTECs by genome-wide identification of T-DMRs. The DNA methylation status at dozens of mTEC-T-DMRs in *Aire*^+/+^ mTECs was similar to that in *Aire*^−/−^ mTECs, while distinct from athymic cells and tissues as well as the other thymic stromal cells, indicating that the Aire-independent DNA methylation profile is involved in differentiation of the mTEC lineage and other thymic stroma cells that form the thymic microenvironment.

## Methods

### Mice

*Aire*^+/−^ mice (B6.129S2-Aire^tm1.1Doi^/J) were obtained from the Jackson Laboratory [22] and backcrossed onto C57BL/6J (B6) mice (Oriental Yeast, Japan). *Aire*^−/−^ mice and their *Aire*^+/+^ littermates were used for isolation of mTECs. The mice were housed and bred under specific pathogen-free conditions.

All experiments using mice were carried out according to the institutional guidelines for the care and use of laboratory animals (Graduate School of Agricultural and Life Sciences, the University of Tokyo).

### Isolation of mTECs

The isolation of mTECs was conducted by modifying previously described methods [16,30]. Briefly, thymi were collected from 6- to 7-week-old male mice, finely minced, washed in RPMI 1640 medium, and sequentially digested twice with 0.2 mg/mL collagenase D for 15 min at 37°C. The pellets were incubated with 0.2 mg/mL collagenase D and 0.2 mg/mL Dispase I (Roche Applied Science) with 25 μg/mL DNase I (Takara), and the reaction was stopped with 5 mM EDTA. Subsequently, after centrifugation at 1350 × *g* on a Percoll gradient, supernatants between ρ = 1.06 g/mL and ρ = 1.0 g/mL were incubated with Mouse BD Fc Block (2.4G2), PE Rat Anti-Mouse CD45 (30-F11) (BD Pharmingen), and FITC-conjugated UEA-1 (Sigma) and sorted using an EPICS Altra flow cytometer (Beckman-Coulter). The fractionation of cells was monitored by RT-PCR (Additional file 10).

### D-REAM analysis

In D-REAM analysis, differential methylation status at HpyCH4IV loci is indicated by differential scores corresponding to fragments generated by this methylation-sensitive restriction enzyme between 2 samples [16; Additional file 2: Figure S2]. In the present study, we performed microarray experiments using mTECs from *Aire*^−/−^ mice and the thymus from C57BL/6J mice. Briefly, after digestion of genomic DNA with HpyCH4IV (New England Biolabs), 50 ng of DNA was ligated with an adaptor, followed by TaqI digestion and ligation with a second adaptor. The fragments were amplified by ligation-mediated PCR to allow selective amplification of unmethylated fragments generated by HpyCH4IV digestion and then hybridized using the GeneChip Mouse Promoter 1.0R Array (Affymetrix), which comprises probes that map to the regions (−6 to +2.5 kb) around the TSSs of approximately 50,000 transcripts (ENST IDs; mm9) obtained from the UCSC genome browser. Hybridization signals were processed with MAT [31] and can be found at Array Express (http://www.ebi.ac.uk/arrayexpress/; accession number: E-MTAB-1113). The data from *Aire*^−/−^ mTECs and the thymus were compared with D-REAM data from the liver, brain, and ES cells [17] to identify fragments that exhibited a difference in microarray scores, thereby indicating the DNA methylation status at corresponding HpyCH4IV sites. We set the cut-off value at 0.5% of FDR (false detecting rates) for T-DMRs. The Galaxy genome browser (https://main.g2.bx.psu.edu/) [32] and “R” (http://www.R-project.org) [33] were used for genomic fragment analysis.

To analyze the gene expression of *Aire*^+/+^ and *Aire*^−/−^ mTECs, we obtained their gene expression profiles from the GEO database (http://www.ncbi.nlm.nih.gov/geo/) [34] and compared with those of the liver and CD4^+^CD8^+^ thymocytes on the same microarray platform (Additional file 5: Table S2). The array data were normalized using the *gcrma* package [35]. The ENST IDs were converted into Affymetrix IDs by using BioMart software (http://www.biomart.org) [36].

### DNA methylation analysis with bisulfite-converted genomic DNA

mTEC cells and gDNAs from the thymus, liver, and ES cells were subjected to bisulfite treatment using the EZ DNA Methylation-Direct Kit (Zymo Research Cooperation) according to the manufacturer’s instructions. Unmethylated cytosine residues are converted into thymine residues by the sodium bisulfite reaction, while methylated cytosine residues remain unchanged. PCR was carried out using the bisulfite-converted genomic DNA with the primers listed in Additional file 11: Table S5. To evaluate the DNA methylation status at specific CpGs, COBRA was performed. The PCR products were evenly divided and incubated with or without HpyCH4IV for 5 h at 37°C and then analyzed with a microchip electrophoresis system (MultiNA, Shimadzu Biotech). DNA methylation levels were calculated as the percentage of digested fragment compared with the sum of digested and undigested fragments. To evaluate the DNA methylation status at CpGs within hundreds of base pairs, the PCR fragments were cloned into the pGEM-T Easy Vector (Promega), and more than 10 clones were sequenced using an ABI 3130 sequencer with a BigDye Terminator kit (Applied Biosystems).

## Abbreviations

mTEC: Medullary thymic epithelial cells; cTEC: Cortical thymic epithelial cells; TRA: Tissue-restricted antigen; Aire: Autoimmune regulator; T-DMR: Tissue-dependent and differentially methylated region; D-REAM: T-DMR profiling with restriction tag-mediated amplification; UEA1: *Ulex europaeus* agglutinin I.

## Competing interests

The authors declare that they have no competing interests.

## Authors’ contributions

GW conceived the study, carried out experiments, analyzed the data, prepared the figures, and drafted the manuscript. KH performed the microarray experiment. SS assisted in bioinformatical analysis of D-REAM data. NA and TA assisted in isolating mTECs and control stromal cells. KS participated in the design of experiments. SY participated in the design of the experiments, performed bioinformatical analysis of D-REAM data, prepared the figures, and drafted the manuscript. All authors read and approved the final manuscript.

## Supplementary Material

Additional file 1 **Figure S1.** Isolation and characterization of CD45^-^UEA1^+^ mTECs and CD45^-^UEA1^-^ thymic stromal cells. Isolation and characterization of CD45^-^UEA1^+^ mTECs and CD45^-^UEA1^-^ thymic stromal cells. A. Isolation of CD45^-^UEA1^+^ mTECs (red) and CD45^-^UEA^-^ thymic stromal cells (yellow) by flow cytometry. Values are representatives of the percentage of each population in repeated isolations. B. Gene expression in mTECs, thymic stromal cells, thymus, liver and ES cells by RT-PCR. Compared with the stromal cells, higher expressions of *Aire*, *Cd80*, *Krt5*, *Krt14*, *Krt8*, *Foxn1* in both the Aire^+/+^ and Aire^−/−^ mTECs were detected. The Aire^−/−^ mice strain used in this research lacks the exon2 of Aire, but the transcription of Aire could be detected [1]. E2, exon2; E3, exon3; E5, exon5. C. Real-time RT-PCR quantification of transcript levels of *Aire* in mTECs isolated from Aire^+/+^ mice, as well as mTEC, thymus from C57BL/6 strain, and ES cells. The Exon2 to Exon3 of *Aire* mRNA was amplified. Standard curve method calculation was used to compute transcript levels relative to *Actb* mRNA. The y axis indicates arbitrary unit normalized to *Actb*, with the expression level in thymus as 1. The expression level of Aire in mTECs was over 50 times higher than in the thymus, which showed a similar pattern as human AIRE in mTECs and the thymus [2]. The expression level of Aire in ES was about half of the thymus. Expression of *Aire* in liver was not detected. Click here for file

Additional file 2 **Figure S2.** Illustration of the D-REAM method for identification of T-DMRs. D-REAM was developed via integration of a methylation-sensitive restriction enzyme (HpyCH4IV), ligation-mediated PCR (LM-PCR), and DNA tiling microarrays [3,4]. Briefly, for a certain genomic locus that contains a HpyCH4IV recognizing site (ACGT), in Sample A, the ACGT containing 5′-methylcytosine cannot be digested by HpyCH4IV and the corresponding fragment is not amplified by LM-PCR, thus does not detected by microarray. In contrast, in Sample B, the ACGT with unmethylated cytocine is digested and the corresponding fragment is efficiently amplified, leading to microarray signal, reflected by D-REAM score. Comparing the two samples using the D-REAM score, the locus is defined as a T-DMR and hypomethylated in Sample B. (PDF 242 kb) Click here for file

Additional file 3 **Table S1.** Summary of mTEC-T-DMRs. (XLSX 12 kb) Click here for file

Additional file 4 **Figure S3.** Distributions of positions relative to TSS of mTECany2-T-DMRs and mTECu-T-DMRs. The highest frequencies of T-DMRs are in the 3′ downstream to TSSs in both mTECany2-T-DMRs and mTECu-T-DMRs. For the histogram, on unit represents 250 bp. (PDF 87 kb) Click here for file

Additional file 5 **Table S2.**List of microarray data of transcriptomes used in this study. Click here for file

Additional file 6 **Figure S4.** Log ratio of the expression levels of genes associated the mTECu-T-DMRs and mTECany2-T-DMRs. Relative expression levels of genes associated with mTECu-T-DMRs (upper) and mTECany2-T-DMRs (lower) in Aire^−/−^ mTECs, Aire^+/+^ mTECs, liver and CD4^+^CD8^+^ thymocytes to Aire^−/−^ mTECs. From left to right, the data sets represented the all probed genes and the T-DMRs, classified according to their relative distance to TSSs as −6 to +2.5 kb (whole window), -1 to +2.5 kb (proximal) and −6 to −1 kb (distal) regions. The vertical axis of the box-plot represents the values of gene expression levels of duplicated data sets relative to one microarray profile of Aire^−/−^ mTECs. The numbers in the round brackets indicate independent gene expression profiles of each type of cell or tissues listed in Table S2. * indicates significant differences in relative expression levels where the *p* values of *t*-tests are less than −0.1. (PDF 128 kb) Click here for file

Additional file 7 **Table S3.** List of mTEC specific genes that associated with mTECu-T-DMRs. (XLSX 12 kb) Click here for file

Additional file 8 **Figure S5.** Genes involved in the development of mTECs with mTEC-T-DMRs. White, mTECu-T-DMR; gray, mTECany2-T-DMR; black, no mTEC-T-DMR. The pathway was modified from Genome Network Platform (http://genomenetwork.nig.ac.jp). *Tnfrsf11a* (RANK) and its down-stream genes *Traf2* and *Lyn* were associated with mTECany2-T-DMRs and *Traf5* was associated with mTECu-T-DMR. TANK (*Tank*), cIAP2 (*Birc2*) and Crinkled (*Edaradd*), which were recruited by TRAF2 to activate the downstream NF-κB [9-11]. Stimulation by RANK leads to activation of STAT1 which in turn up-regulates IFN-stimulated gene expression in mTECs [12]. *Stat1* and *Jak1* were found to be associated with mTECu-T-DMR and mTECany2-T-DMR, respectively. (PDF 82 kb) Click here for file

Additional file 9 **Table S4.** T-DMRs analyzed by COBRA. Click here for file

Additional file 10 Methods and references to additional files. Click here for file

Additional file 11 **Table S5.**List of primers for bisulfite PCR. Click here for file
