# Supplementary figures and images for: DNA methylation profile of Aire-deficient mouse medullary thymic epithelial cells
Source: BMC Immunol. 2012 Nov 2;13:58. doi: 10.1186/1471-2172-13-58 (PMC3546423; doi:10.1186/1471-2172-13-58)

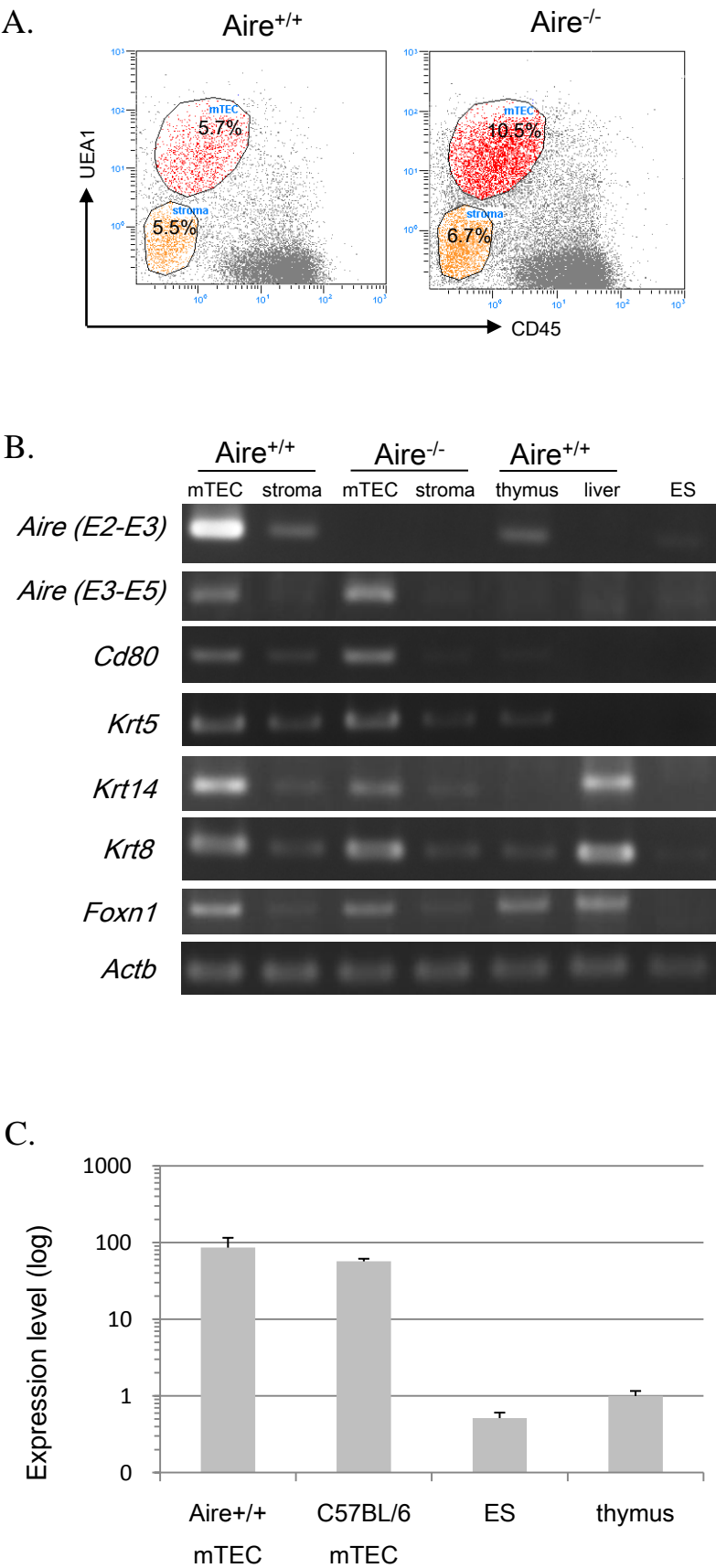

Supplement: Additional file 1 — Figure S1. Isolation and characterization of CD45-UEA1+ mTECs and CD45-UEA1- thymic stromal cells. Isolation and characterization of CD45-UEA1+ mTECs and CD45-UEA1- thymic stromal cells. A. Isolation of CD45-UEA1+ mTECs (red) and CD45-UEA- thymic stromal cells (yellow) by flow cytometry. Values are representatives of the percentage of each population in repeated isolations. B. Gene expression in mTECs, thymic stromal cells, thymus, liver and ES cells by RT-PCR. Compared with the stromal cells, higher expressions of Aire, Cd80, Krt5, Krt14, Krt8, Foxn1 in both the Aire+/+ and Aire−/− mTECs were detected. The Aire−/− mice strain used in this research lacks the exon2 of Aire, but the transcription of Aire could be detected [1]. E2, exon2; E3, exon3; E5, exon5. C. Real-time RT-PCR quantification of transcript levels of Aire in mTECs isolated from Aire+/+ mice, as well as mTEC, thymus from C57BL/6 strain, and ES cells. The Exon2 to Exon3 of Aire mRNA was amplified. Standard curve method calculation was used to compute transcript levels relative to Actb mRNA. The y axis indicates arbitrary unit normalized to Actb, with the expression level in thymus as 1. The expression level of Aire in mTECs was over 50 times higher than in the thymus, which showed a similar pattern as human AIRE in mTECs and the thymus [2]. The expression level of Aire in ES was about half of the thymus. Expression of Aire in liver was not detected. [file 1471-2172-13-58-S1.pdf]

Genome-wide identification of genomic loci with T-DMRs by D-REAM

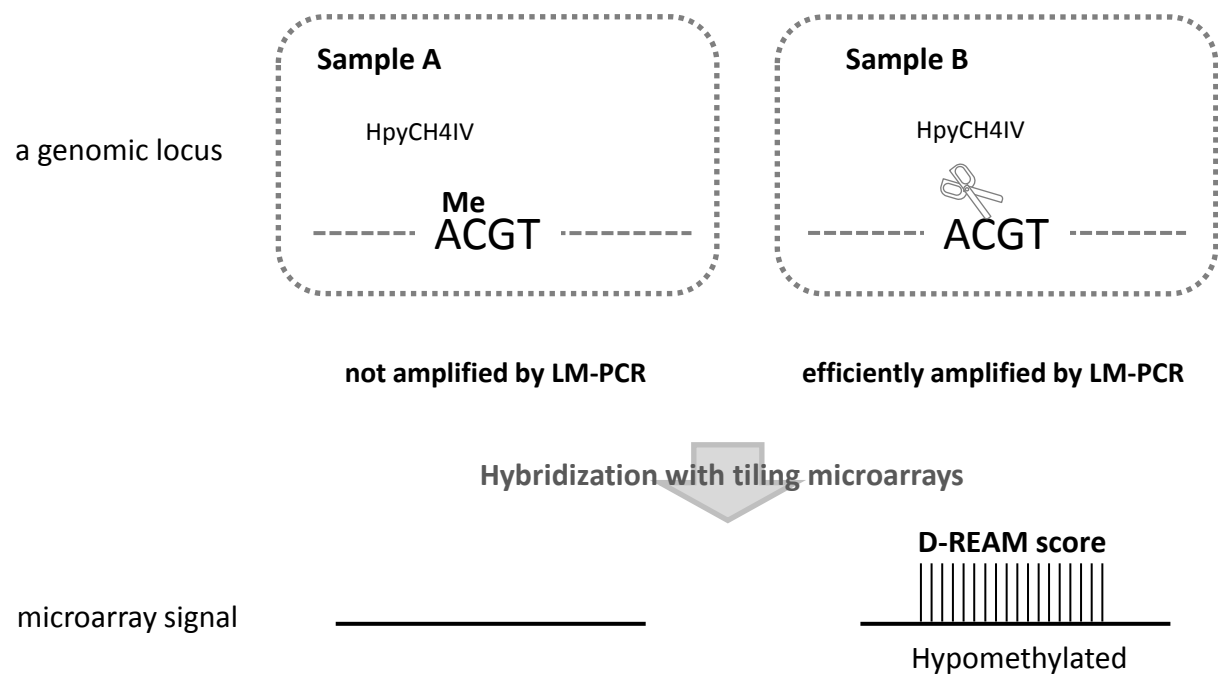

Supplement: Additional file 2 — Figure S2. Illustration of the D-REAM method for identification of T-DMRs. D-REAM was developed via integration of a methylation-sensitive restriction enzyme (HpyCH4IV), ligation-mediated PCR (LM-PCR), and DNA tiling microarrays [3,4]. Briefly, for a certain genomic locus that contains a HpyCH4IV recognizing site (ACGT), in Sample A, the ACGT containing 5′-methylcytosine cannot be digested by HpyCH4IV and the corresponding fragment is not amplified by LM-PCR, thus does not detected by microarray. In contrast, in Sample B, the ACGT with unmethylated cytocine is digested and the corresponding fragment is efficiently amplified, leading to microarray signal, reflected by D-REAM score. Comparing the two samples using the D-REAM score, the locus is defined as a T-DMR and hypomethylated in Sample B. (PDF 242 kb) [file 1471-2172-13-58-S2.pdf]

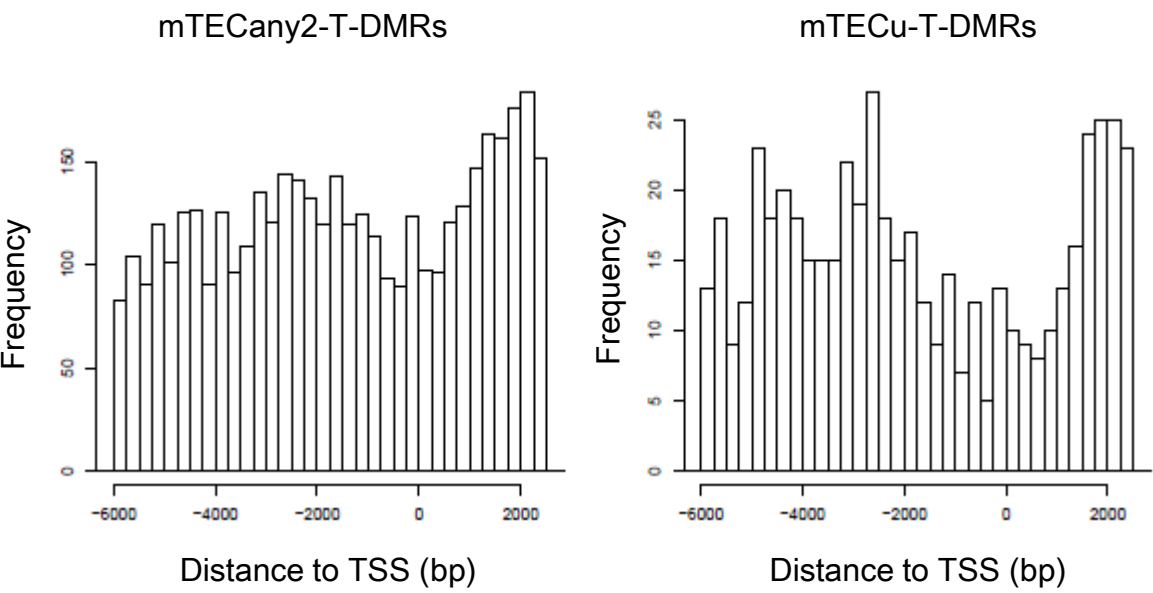

Supplement: Additional file 4 — Figure S3. Distributions of positions relative to TSS of mTECany2-T-DMRs and mTECu-T-DMRs. The highest frequencies of T-DMRs are in the 3′ downstream to TSSs in both mTECany2-T-DMRs and mTECu-T-DMRs. For the histogram, on unit represents 250 bp. (PDF 87 kb) [file 1471-2172-13-58-S4.pdf]

Additional File 8. Wu *et al.*  
Figure S5.

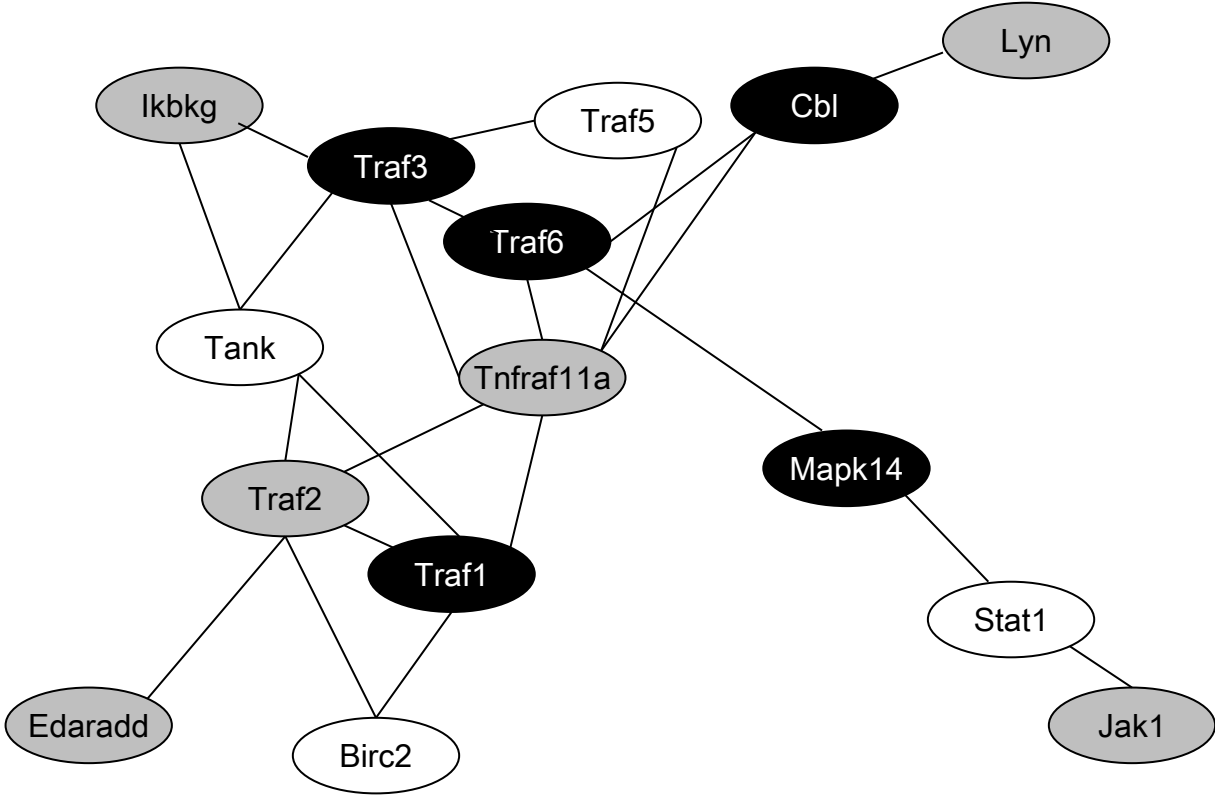

Supplement: Additional file 8 — Figure S5. Genes involved in the development of mTECs with mTEC-T-DMRs. White, mTECu-T-DMR; gray, mTECany2-T-DMR; black, no mTEC-T-DMR. The pathway was modified from Genome Network Platform (http://genomenetwork.nig.ac.jp). Tnfrsf11a (RANK) and its down-stream genes Traf2 and Lyn were associated with mTECany2-T-DMRs and Traf5 was associated with mTECu-T-DMR. TANK (Tank), cIAP2 (Birc2) and Crinkled (Edaradd), which were recruited by TRAF2 to activate the downstream NF-κB [9-11]. Stimulation by RANK leads to activation of STAT1 which in turn up-regulates IFN-stimulated gene expression in mTECs [12]. Stat1 and Jak1 were found to be associated with mTECu-T-DMR and mTECany2-T-DMR, respectively. (PDF 82 kb) [file 1471-2172-13-58-S8.pdf]
