# Supplementary material for: DNA methylation profile of Aire-deficient mouse medullary thymic epithelial cells
Source: BMC Immunol. 2012 Nov 2;13:58. doi: 10.1186/1471-2172-13-58 (PMC3546423; doi:10.1186/1471-2172-13-58)

Additional File 6. Wu *et al.*
Figure S4

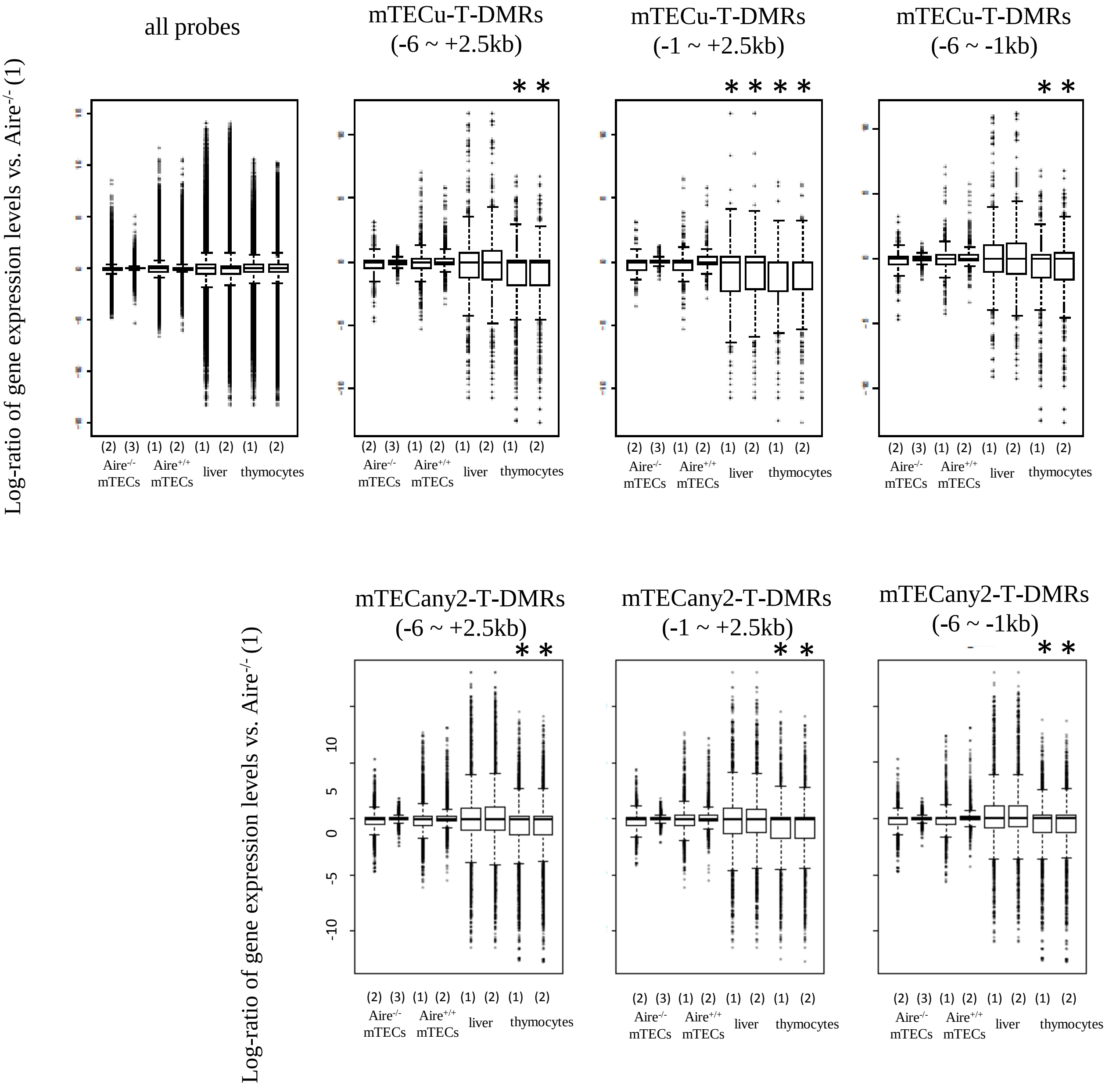

Supplement: Additional file 6 — Figure S4. Log ratio of the expression levels of genes associated the mTECu-T-DMRs and mTECany2-T-DMRs. Relative expression levels of genes associated with mTECu-T-DMRs (upper) and mTECany2-T-DMRs (lower) in Aire−/− mTECs, Aire+/+ mTECs, liver and CD4+CD8+ thymocytes to Aire−/− mTECs. From left to right, the data sets represented the all probed genes and the T-DMRs, classified according to their relative distance to TSSs as −6 to +2.5 kb (whole window), -1 to +2.5 kb (proximal) and −6 to −1 kb (distal) regions. The vertical axis of the box-plot represents the values of gene expression levels of duplicated data sets relative to one microarray profile of Aire−/− mTECs. The numbers in the round brackets indicate independent gene expression profiles of each type of cell or tissues listed in Table S2. * indicates significant differences in relative expression levels where the p values of t-tests are less than −0.1. (PDF 128 kb) [file 1471-2172-13-58-S6.pdf]
