# Supplementary material for: DNA methylation profile of Aire-deficient mouse medullary thymic epithelial cells
Source: BMC Immunol. 2012 Nov 2;13:58. doi: 10.1186/1471-2172-13-58 (PMC3546423; doi:10.1186/1471-2172-13-58)
Supplement: Additional file 10 — Methods and references to additional files. [file 1471-2172-13-58-S10.docx]

**Additional File 10- as DOC.**

## **Methods**

**RNA extraction, reverse transcription, and real-time PCR**

Total RNA from the Aire^+/+^ and Aire^-/-^ mTECs, stromal cells, liver, thymus and ES cells were extracted using Trizol Reagent. Before synthesis of first-strand cDNA, the RNA preparation was treated with RNase-free DNase I (Takara) to eliminate any residual genomic DNA. The total RNA was then reverse-transcribed using Olig(dT) and SuperScript III First-Strand Synthesis System (Invitrogen) according to the manufacturer’s instructions. The synthesized cDNA was subjected to PCR using following primers:

Aire_RT_E2F CATCCTGGATTTCTGGAGGATT

Aire_RT_E3R CTCCTCCAGTGCTTTTCTCTTG

Aire_Exon3F GACCTAAACCAGTCCCGGAA

Aire_Exon5R ATCCCTTCCACGGCCCCT

Cd80_RT_F AGTAATAACAGTCGTCGTCATCGTT

Cd80_RT_R ATGAGCCACATAATACCATGTATCC

Krt5_RT_F CTGGAGGCATCCAAGAGGT

Krt5_RT_R CGGATCCAGGTTCTGCTTTA

Krt8_RT_F CAGGAGAAGCTGAAGCTGG

Krt8_RT_R CGGAGGAAGTTGATCTCGTC

Krt14_RT_F GATGTGAAGACAAGGCTGGAG

Krt14_RT_R CATGTAGCAGCTTTAGTTCTTGGTG

Foxn1_RT_F CCCAGGACTCACCTCTACCTG

Foxn1_RT_R GAGTAGAGAGTGCTGAGGTGCAT

Actb_F TTCTACAATGAGCTGCGTGTGG

Actb_R ATGGCTGGGGTGTTGAAGGT

Real-time PCR was performed in triplicates using the Quantitect SYBR Green PCR kit (Qiagen) with ABI 7500 Real Time PCR system (Applied Biosystems).

# **References**

1. Anderson MS, Venanzi ES, Klein L, Chen Z, Berzins SP, Turley SJ, von Boehmer H, Bronson R, Dierich A, Benoist C *et al*: **Projection of an immunological self shadow within the thymus by the aire protein.** *Science* 2002, **298**(5597):1395-1401.
2. Klamp T, Sahin U, Kyewski B, Schwendemann J, Dhaene K, Türeci O: **Expression profiling of autoimmune regulator AIRE mRNA in a comprehensive set of human normal and neoplastic tissues**. *Immunol Lett* 2006, **106**(2):172-179.
3. Yagi S, Hirabayashi K, Sato S, Li W, Takahashi Y, Hirakawa T, Wu G, Hattori N, Ohgane J, Tanaka S *et al*: **DNA methylation profile of tissue-dependent and differentially methylated regions (T-DMRs) in mouse promoter regions demonstrating tissue-specific gene expression.** *Genome Res* 2008, **18**(12):1969-1978.
4. Sato S, Yagi S, Arai Y, Hirabayashi K, Hattori N, Iwatani M, Okita K, Ohgane J, Tanaka S, Wakayama T *et al*: **Genome-wide DNA methylation profile of tissue-dependent and differentially methylated regions (T-DMRs) residing in mouse pluripotent stem cells.** *Genes Cells* 2010, **15**(6):607-618.
5. Venanzi ES, Melamed R, Mathis D, Benoist C: **The variable immunological self: genetic variation and nongenetic noise in Aire-regulated transcription.** *Proc Natl Acad Sci U S A* 2008, **105**(41):15860-15865.
6. Lattin JE, Schroder K, Su AI, Walker JR, Zhang J, Wiltshire T, Saijo K, Glass CK, Hume DA, Kellie S *et al*: **Expression analysis of G Protein-Coupled Receptors in mouse macrophages**. *Immunome Res* 2008, **4**:5.
7. Johnnidis JB, Venanzi ES, Taxman DJ, Ting JP, Benoist CO, Mathis DJ: **Chromosomal clustering of genes controlled by the aire transcription factor.** *Proc Natl Acad Sci U S A* 2005, **102**(20):7233-7238.
8. Derbinski J, Gäbler J, Brors B, Tierling S, Jonnakuty S, Hergenhahn M, Peltonen L, Walter J, Kyewski B: **Promiscuous gene expression in thymic epithelial cells is regulated at multiple levels.** *J Exp Med* 2005, **202**(1):33-45.
9. Pomerantz JL, and Baltimore D: **NF-kappaB activation by a signaling complex containing TRAF2, TANK and TBK1, a novel IKK-related kinase.** *EMBO J* 1999. **18**(23): 6694-6704.
10. Vince JE, Pantaki D, Feltham R., Mace PD, Cordier SM, Schmukle AC, Davidson AJ *et al.*, **TRAF2 must bind to cellular inhibitors of apoptosis for tumor necrosis factor (tnf) to efficiently activate nf-{kappa}b and to prevent tnf-induced apoptosis.** *J Biol Chem* 2009. **284**(51): 35906-35915.
11. Yan M, Zhang Z, Brady JR, Schilbach S, Fairbrother WJ and Dixit VM: **Identification of a novel death domain-containing adaptor molecule for ectodysplasin-A receptor that is mutated in crinkled mice.** *Curr Biol* 2002. **12**(5): 409-413.
12. Ohshima D, Qin J, Konno H, Hirosawa A, Shiraishi T, Yanai H, Shimo Y *et al.*: **RANK signaling induces interferon-stimulated genes in the fetal thymic stroma.** *Biochem Biophys Res Commun* 2011. **408**(4): 530-536.
